# Felty Syndrome in a Patient Presenting With Bilateral Scleritis and Multiple Autoimmune Syndrome: A Case Report

**DOI:** 10.7759/cureus.57468

**Published:** 2024-04-02

**Authors:** Cameron Rattray, Sowmya Dandu, Mohammad A Hossain

**Affiliations:** 1 Internal Medicine, St. George's University School of Medicine, St. George, GRD; 2 Internal Medicine, Jersey Shore University Medical Center, Neptune City, USA; 3 Medicine, Hackensack Meridian School of Medicine, Nutley, USA

**Keywords:** multiple autoimmune syndrome, neutropenia, splenomegaly, sle, rheumatoid arthritis, felty syndrome

## Abstract

Autoimmune diseases can result in additional symptoms and complications impacting various organ systems beyond the joints. These can affect the eyes, skin, respiratory, cardiac, and renal systems. Recognizing and understanding these diverse manifestations, such as the severe eye issues seen in rheumatoid arthritis (RA) and the potentially life-threatening Felty syndrome, is crucial for clinicians to promptly identify and treat these conditions effectively.

In this case presentation, we report on a patient admitted for bilateral scleritis, which was found to be secondary to multiple autoimmune syndrome type 3. During the patient's hospital stay, Felty syndrome was incidentally diagnosed due to the observed combination of RA, splenomegaly, and absolute neutropenia. Prompt recognition of this condition allowed the patient to receive appropriate care, including oral steroids, hydroxychloroquine, and methotrexate, decreasing the risk of severe complications.

## Introduction

Autoimmune diseases can lead to various extra-articular manifestations and complications affecting different organ systems, including the eyes, skin, respiratory, cardiac, and renal systems. Multiple autoimmune syndrome (MAS) is described as the coexistence of three or more autoimmune diseases, the classification of which is defined by the prevalence of a particular autoimmune disease being associated with two other autoimmune diseases [1,2]. The classification includes the following: (I). Type 1 MAS encompasses myasthenia gravis, thymoma, polymyositis, and giant cell myocarditis [1,2]. (II). Type 2 MAS includes Sjögren's syndrome, rheumatoid arthritis (RA), primary biliary cirrhosis (PBC), scleroderma, and autoimmune thyroid disease [1,2]. (III). Type 3 MAS comprises autoimmune thyroid disease, myasthenia gravis and/or thymoma, Sjögren's syndrome, pernicious anemia, idiopathic thrombocytopenic purpura (ITP), Addison's disease, type 1 diabetes mellitus, vitiligo, autoimmune hemolytic anemia (AIHA), systemic lupus erythematosus (SLE), and dermatitis herpetiformis [1,2].

Ophthalmologic manifestations of autoimmune diseases, particularly in a multi-disease setting, are a common occurrence, and those of RA can be particularly severe, including conditions like scleritis and episcleritis [3]. Scleritis, in particular, can also be a complication of other autoimmune diseases, such as Sjogren's syndrome. Sjogren's syndrome, which is closely associated with SLE and RA, falls under the category of MAS type 3 [1].

Among these ophthalmologic complications, scleritis is a highly severe condition that often requires hospitalization to prevent vision loss or the affected eye's loss. However, there is an even rarer and more dangerous complication of RA known as Felty syndrome. This syndrome increases patients' risk of severe and recurrent infections. Felty syndrome is characterized by the triad of RA, splenomegaly (enlarged spleen), and absolute neutropenia [3,4].

Felty syndrome, though rare, poses a substantial threat to patients and requires prompt and attentive medical management. The compromised immune function caused by absolute neutropenia can lead to life-threatening infections [4], making early detection and appropriate treatment vital for patients with this condition.

In this case study, we present a patient admitted for bilateral scleritis associated with MAS type 3. Throughout the patient's hospitalization, an incidental diagnosis of Felty syndrome was established based on the concurrent presence of RA, splenomegaly, and absolute neutropenia.

Understanding the various extra-articular manifestations and complications of autoimmune diseases, such as the severe ophthalmologic manifestations in RA and the life-threatening Felty syndrome, can aid clinicians in promptly recognizing and managing these conditions.

## Case presentation

A 46-year-old female with a medical history significant of RA, Sjogren's syndrome, and SLE presented to the emergency department with severe and progressively worsening pain in her right eye for five days, accompanied by blurry vision and photophobia, as well as a dull headache on the right side for 10 days. She did not report fever, nausea, vomiting, unintentional weight loss, abnormal skin pigmentation, increased bleeding, or worsening fatigue.

During the physical examination, bilateral congested scleral vessels and conjunctival hyperemia were observed, with the right eye being more affected. Additionally, swan neck deformities were noted in the fingers on both hands. The patient's vital signs were within normal limits, and no other significant findings were observed.

Laboratory findings were significant for lymphocytopenia of 16.3% with absolute leukopenia and absolute neutropenia, iron deficiency anemia, hypoalbuminemia with normal calcium (8.9 mg/dL; when corrected for albumin), mildly elevated erythrocyte sedimentation rate and C-reactive protein, and decreased serum complement factors, C3 and C4 (Table 1).

**Table 1:** Abnormal laboratory findings

Laboratory finding	Value
Lymphocytopenia	16.3%
Leukocytes	1.9x10^3/uL
Neutrophils	0.8x10^3/uL
Hemoglobin	9.3 g/dL
Hematocrit	32.7%
Mean corpuscular volume (MCV)	71.6 fL
RDW (red cell distribution width)	16.8%
Serum Iron	20 ug/dL
Transferrin	249 mg/dL
Albumin	3.1 g/dL
Calcium (corrected for albumin)	8.9 mg/dL
C-reactive protein	1.11 mg/dL
Serum C3	69 mg/dL
Serum C4	5 mg/dL

Upon ophthalmologic evaluation with a slit lamp (Table 2) and dilated fundus examination, the patient was diagnosed with right eye scleritis and left eye episcleritis. The cup/disc ratio on dilated fundus examination was found to be 0.2, with the vitreous humor, macula, blood vessels, and peripheral structures appearing normal in both eyes. Subsequently, she was admitted to the inpatient wards and initiated on IV methylprednisone 40 mg/q8h, hydroxychloroquine 200 mg/BID, and naproxen 500 mg/BID.

**Table 2:** Portable slit lamp examination findings

Slit lamp anatomy	Findings
Lids/ocular adnexa	No abnormality detected
Conjunctiva/sclera	2+ scleritis inferior nasal right eye, 1+ episcleritis nasally left eye
Cornea	Extensive inferior scarring right eye, pterygium, and moderate epitheliopathy in both eyes
Anterior chamber	No abnormality detected
Pupil	Round, regular, and reacting to light bilaterally
Lens	Clear bilaterally

**Figure 1:**
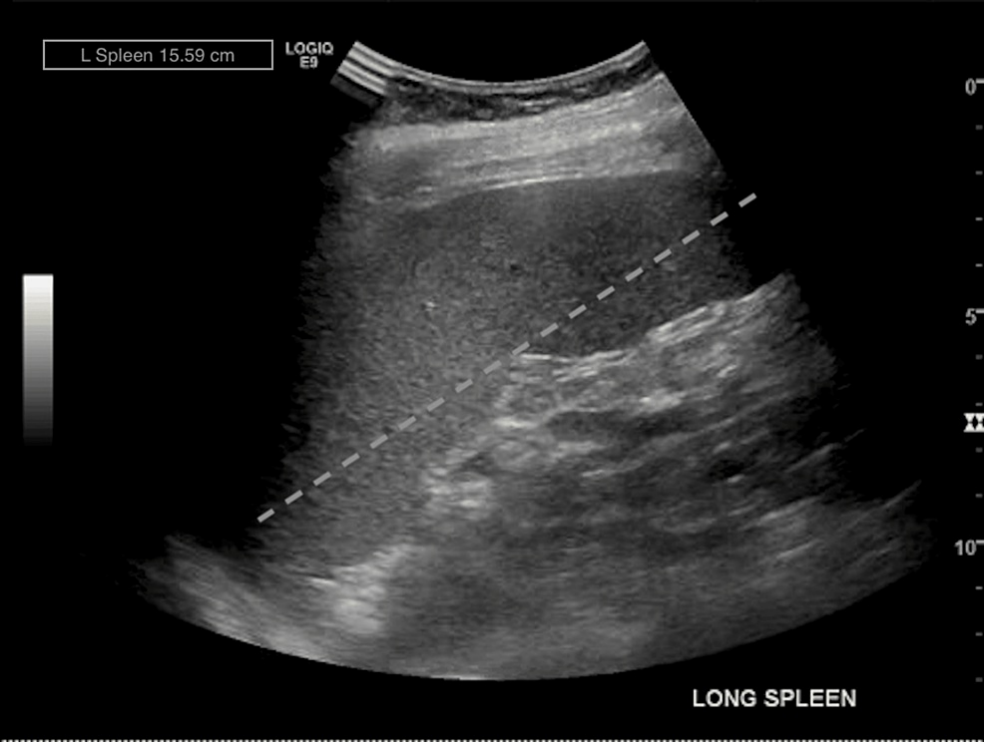
Longitudinal ultrasound of spleen

**Figure 2:**
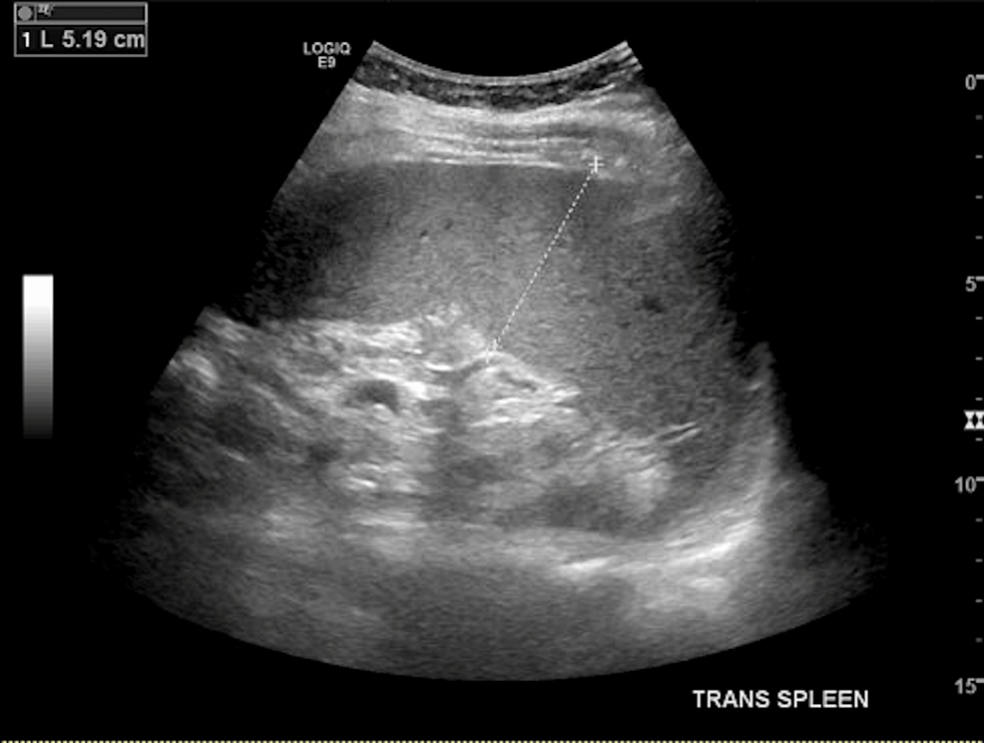
Transverse ultrasound of spleen

Due to the patient's MASs and the development of scleritis, a rheumatology consultation was completed. Provided the patient's worsening anemia from baseline and leukopenia with absolute neutropenia, a splenic ultrasound was recommended and completed (Figures 1, 2). The ultrasound identified splenomegaly, measuring 15.1 cm by 5.19 cm (normal dimensions: 11-12 cm by 3-4 cm), compared to prior abdominal ultrasounds. Given the historical diagnosis of RA and the new findings of absolute leukopenia, absolute neutropenia, and splenomegaly, the patient was diagnosed with Felty syndrome.

Six months before this admission, an investigational bone marrow biopsy had been performed for chronic leukopenia, proving trilineage hematopoiesis with eosinophilia and polyclonal plasmacytosis, most likely associated with extensive autoimmune disease. No immunophenotypic abnormalities were detected, ruling out plasma cell dyscrasias, leukemias, or lymphoproliferative disorders at that time, which supports Felty syndrome as the likely cause for the alterations in serum absolute leukocytes and neutrophils on this admission.

Following the diagnosis, the patient continued to receive IV methylprednisolone 1000 mg/day until ophthalmology and rheumatology medically cleared her for discharge. Subsequently, she was switched to an oral prednisone taper and maintained on hydroxychloroquine 200 mg twice a day. Upon discharge, she was instructed to follow up as an outpatient regarding her new diagnosis of Felty syndrome.

During her outpatient follow-up two weeks later, the scleritis in both eyes had significantly improved. As a result, methotrexate 10 mg/weekly was initiated. Repeated lab tests were requested to monitor the progression or remission of the patient's abnormal lab findings from her hospital admission. Her medications from discharge were continued as prescribed, with 10 mg daily of prednisone, 200 mg twice a day of hydroxychloroquine, and methotrexate 10 mg weekly. Additionally, the patient received counseling on the therapeutic challenge of neutropenia and was advised on proper hygiene and infection prevention.

## Discussion

MAS refers to the occurrence of three or more autoimmune diseases concurrently, a phenomenon observed in up to 25% of individuals with autoimmune conditions [1,2]. The existing literature describes three types of MAS: type 1, type 2, and type 3. Type 1 MAS includes myasthenia gravis, thymoma, polymyositis, and giant cell myocarditis. Type 2 MAS comprises Sjögren's syndrome, RA, PBC, scleroderma, and autoimmune thyroid disease. Type 3 MAS involves a combination of autoimmune thyroid disease, myasthenia gravis, thymoma, Sjögren's syndrome, pernicious anemia, ITP, Addison's disease, type 1 diabetes mellitus, vitiligo, AIHA, SLE, and dermatitis herpetiformis. Based on this classification, our patient falls into the type 3 MAS category [1,2].

Sjogren’s syndrome is a systemic autoimmune disease frequently associated with SLE and RA, as evident in our patient [1]. The management and treatment of MAS present challenges, given the multitude of potential complications that often necessitate a multidisciplinary approach involving various pharmacological and surgical interventions to prevent disease progression.

Both RA and Sjogren’s syndrome carry individual risks for developing episcleritis and scleritis [3]. When occurring together, they further elevate the risk of these ocular complications. Episcleritis and scleritis are the most common ophthalmologic inflammatory findings in RA, with 2% of patients developing these conditions [5]. These conditions may be even more severe complications for patients with Sjogren's syndrome. Episcleritis is generally benign and can be successfully treated with non-steroidal anti-inflammatory drugs and/or topical steroids. On the other hand, scleritis often necessitates disease-modifying anti-rheumatic drugs (DMARDs) for effective management [3].

RA, SLE, and Sjogren’s syndrome can all lead to various complications, with scleritis being recognized as a severe complication that, in extreme cases, can result in vision loss or loss of the eye [3]. However, in the case of our patient, the subsequent abnormal laboratory workup raised suspicion of a rare and dangerous complication of RA: Felty syndrome.

Felty syndrome is a rare extra-articular manifestation of seropositive RA characterized by the triad of RA, absolute neutropenia, and splenomegaly. Due to the increased risk of severe and recurrent infections, Felty syndrome poses a significant threat to patients with RA, primarily attributed to absolute neutropenia. While all three manifestations mentioned above are typical, the diagnosis of Felty syndrome may still be considered if neutropenia is present along with splenomegaly. However, neutropenia with fewer than 2000 cells/mL must be evident [1,4]. Other findings consistent with Felty syndrome include anemia, thrombocytopenia, positive rheumatoid factor, anti-cyclic citrullinated peptide antibodies, antinuclear antibodies, and anti-histone antibodies. In bone marrow biopsy, myeloid hyperplasia with increased granulopoiesis may be observed, along with venous sinusoid congestion, polyclonal plasmacytosis, and reticular cell and germinal cell hyperplasia in splenic biopsy [6].

Typically, Felty syndrome presents symptomatically with fever, weight loss, alterations in skin pigmentation, and abnormal fatigue [4,5]. However, none of these symptoms were present in our reported patient upon admission. Moreover, while the patient presented with bilateral scleritis, no evidence of an infectious process was noted during the physical exam or laboratory workup.

Currently, no randomized control trials exist for the treatment of Felty syndrome. Low-dose methotrexate is considered the first-line treatment due to its demonstrated efficacy in improving neutrophil count within four to six weeks [4,6,7]. The effect of methotrexate is dose-dependent and noticeable changes may be observed within four to eight weeks, thus necessitating an adequate trial with the maximum tolerated dose before determining the neutropenia as unresponsive to methotrexate [4]. Our patient is currently being trialed with a dosage of 10 mg/week, 2.5 mg/week higher than the dosage shown to improve neutrophil count within four to six weeks in observational studies.

Other agents, such as leflunomide, parenteral gold therapy, cyclosporine, and rituximab, have also shown benefits in the treatment of Felty syndrome [4,6-8]. Leflunomide has significantly improved neutropenia in patients with Felty syndrome, while parenteral gold therapy and cyclosporine are no longer preferred due to their adverse event profiles [4,8]. Rituximab has been shown to improve neutrophil count without significant side effects, particularly in patients who fail to respond or cannot tolerate non-biologic DMARD therapies [9]. Additionally, although glucocorticoids have historically been used in the treatment of Felty syndrome, long-term use is not typically advised due to the significant immunosuppressive effects and possible worsening of underlying autoimmune conditions [4,6]. Finally, for patients with absolute neutrophil counts of less than 1000 cells/mL with severe and recurrent infections, who fail to respond to DMARDs and rituximab, granulocyte colony-stimulating factor (G-CSF) is a pharmacological intervention that has been shown to significantly increase neutrophil count within one week of treatment [4,6,9].

Splenectomy has been pursued with some success in patients with Felty syndrome; however, this surgical intervention is usually reserved for cases where all pharmacological interventions, including DMARDs and biologic agents like rituximab and G-CSF, have failed [4].

Data on the prognosis of patients with Felty syndrome is currently limited. However, advanced treatment options like biologic agents and G-CSF have improved the severity and extra-articular manifestations of RA, reducing the need for invasive splenectomy [4].

In our patient's case, there were no signs of infection, and the detection of Felty syndrome was incidental. With the addition of first-line therapy, methotrexate, to the patient’s treatment regimen, close monitoring will be conducted to assess the response to treatment and the appearance of any infections.

## Conclusions

Our patient was incidentally diagnosed with Felty syndrome during the diagnostic workup for bilateral scleritis, secondary to MAS type 3, explicitly involving RA and Sjogren’s syndrome.

This case underscores the significance of a comprehensive diagnostic approach to RA and MAS, as it can help anticipate devastating complications like those associated with Felty syndrome. Moreover, it emphasizes the need for more randomized controlled trials and observational studies to determine the optimal treatment for Felty syndrome and establish the prognosis for patients affected by this condition.
